# Impact of environmental changes on the behavioral diversity of the Odonata (Insecta) in the Amazon

**DOI:** 10.1038/s41598-021-88999-7

**Published:** 2021-05-07

**Authors:** Bethânia O. de Resende, Victor Rennan S. Ferreira, Leandro S. Brasil, Lenize B. Calvão, Thiago P. Mendes, Fernando G. de Carvalho, Cristian C. Mendoza-Penagos, Rafael C. Bastos, Joás S. Brito, José Max B. Oliveira-Junior, Karina Dias-Silva, Ana Luiza-Andrade, Rhainer Guillermo, Adolfo Cordero-Rivera, Leandro Juen

**Affiliations:** 1 Laboratório de Ecologia e Conservação, Instituto de Ciências Biológicas, Universidade Federal do Pará, Belém, Brazil; 2 Graduate Program in Ecology-PPGECO, Universidade Federal do Pará, Belém, Brazil; 3 Instituto de Ciências e Tecnologia das Águas (ICTA), Universidade Federal do Oeste do Pará (UFOPA), Rua Vera Paz, s/n (Unidade Tapajós) Bairro Salé, Santarém, Pará 68040-255 Brazil; 4 LESTES Lab, DHb, UFSCar, São Carlos, São Paulo Brazil; 5 Universidade de Vigo, ECOEVO Lab, EE Forestal, Campus Universitario A Xunqueira, 36005 Pontevedra, Spain; 6 Graduate Program in Agriculture and the Environment-PPGAA, Universidade Estadual do Maranhão, Balsas, Maranhão Brazil; 7 Graduate Program in Environmental Sciences-PPGCA, Universidade Federal do Amapá, Macapá, Amapá Brazil

**Keywords:** Behavioural ecology, Ecology

## Abstract

The odonates are insects that have a wide range of reproductive, ritualized territorial, and aggressive behaviors. Changes in behavior are the first response of most odonate species to environmental alterations. In this context, the primary objective of the present study was to assess the effects of environmental alterations resulting from shifts in land use on different aspects of the behavioral diversity of adult odonates. Fieldwork was conducted at 92 low-order streams in two different regions of the Brazilian Amazon. To address our main objective, we measured 29 abiotic variables at each stream, together with five morphological and five behavioral traits of the resident odonates. The results indicate a loss of behaviors at sites impacted by anthropogenic changes, as well as variation in some morphological/behavioral traits under specific environmental conditions. We highlight the importance of considering behavioral traits in the development of conservation strategies, given that species with a unique behavioral repertoire may suffer specific types of extinction pressure.

## Introduction

The enormous variety of behavior exhibited by most animals has inspired human thought, arts, and Science for centuries, from rupestrian paintings to the Greek philosophers. One prominent group of animals, the insects, present a wide range of complex behaviors^1,2^, mostly related to reproduction, such as elaborate courtship rituals, and stereotyped territorial and mating behaviors^3,4^. Over the years, a large body of research has sought to identify and describe the evolutionary and ecological processes that have created and maintained the myriad of behavioral patterns found among the different insect groups^5^.

The insects of the order Odonata are good models for the assessment of ecological questions on animal behavior, in particular because of their diverse reproductive modes and mating strategies^6,7^. These diverse behaviors include territoriality in many species, which is usually associated with mating and oviposition sites^8^. The oviposition behavior of odonates can be classified in three main types: (1) exophytic, when the female lays eggs directly in the water, usually touching the surface a number of times while hovering; (2) endophytic, when the female lays eggs inside the living tissue of plants, and (3) epiphytic, when the female oviposits on exposed surfaces, such as roots, debris, moss, phytotelmata or even the ground or rocks^6^. Odonate territories may vary considerably in size, from a few square centimeters to many square meters, and may contain a range of valuable resources, which include sunning spots, perches, and oviposition substrates^9,10^. In many species, dominant males can often be observed patrolling their territories, and these individuals tend to copulate within the area or relatively close to their territory^11^. Odonate males may also present agonistic behavior, settling territorial disputes through physical aggression or non-contact aerial displays, flashing their wings toward intruders or chasing rivals away^9,12^.

Many odonates present specific mating behaviors, and some species engage in courtship, with the males courting the females prior to mating^13^. For example, the males of *Calopteryx xanthostoma* (Charpentier, 1825) and *Calopteryx haemorrhoidalis* Vander Linden, 1825, perform elaborate flights, dropping to the water and floating with the current to demonstrate the oviposition site to the potential female mate^14^, or extend their legs and iridescent wings toward the female^15^. After mating, the males of some species may exhibit mate-guarding strategies, which are typically categorized as: (1) contact guarding, when the male remains in the tandem position (or perched directly on the female) during oviposition; (2) non-contact guarding, when the male perches or hovers near the female during oviposition, and (3) no mate-guarding, when the female oviposits alone, without the presence of the male^16,17^.

The enormous behavioral diversity of the Odonata, the conspicuity of the males in the field, and the favorable conditions for the collection of behavioral data combine to make this insect order an excellent model for comparative studies^6^. Despite this, little is known about the behavior of most South American species, and data are especially scant for the species from the Amazon region, which are in constant threat from anthropic actions^18^. The ongoing increase in the modification of natural landscapes has raised concerns among researchers with regard to the loss of or changes to behavioral traits, in particular those related to reproduction^19–21^. Environmental alterations may affect both mating behavior and habitat selectivity, which may ultimately alter community structure, influencing species richness, and their abundance and distribution^19,20^. In addition to the recent discussion of the need to conserve ethodiversity and behavioral repertoires, a range of studies have focused on the effects of modifications in the landscape on local animal communities and their associated behavioral patterns, with this ethological focus now being considered a major ally of biodiversity conservation programs^22^.

Caro & Sherman^23^ and Harabiš et al.^24^ demonstrated that many odonate taxa have behavioral traits that are highly sensitive to local ecological conditions, and that the characteristics of the environment are fundamental to the structuring of odonate communities^25^. We predicted that odonate behavioral diversity will be equally vulnerable to environmental change. Given this, the principal objective of the present study was to assess the effects of environmental alterations provoked by shifts in land use on the behavioral diversity of adult odonates of the suborders Zygoptera and Anisoptera. We assume that the environmental modifications caused by shifts in land use alter the behavioral diversity of both odonate suborders^26^. In particular, these changes may lead to the exclusion of species with specific habits that are dependent on a given type of microhabitat^27^. Given this, we would predict a greater overall behavioral richness (i.e., a larger number of different types of behavior) in the zygopterans, and greater behavioral evenness in preserved areas, where the more favorable resource availability may allow for more univariate niche overlap. In altered areas, by contrast, we would expect an increase in niche differentiation, to allow species to coexist. In these areas, we would predict that the removal of the riparian vegetation and the more open forest canopy of the streams will lead, in particular, to the loss of behaviors associated with specific types of substrate, such as oviposition sites and perches^28,29^. In the case of the anisopterans, we would predict the opposite pattern, due to the ecophysiological differences between the two suborders^6,25,30^, with greater behavioral richness and evenness in altered areas, and more behavioral divergence in more preserved areas. We predict specifically that areas with higher deforestation rates and more open canopies will be associated with the establishment of more generalist species, such as those of the genera *Orthemis* Hagen, 1861 and *Erythrodiplax* Brauer, 1868, and an increase in the overall behavioral repertoire of the different species found in these areas, albeit with increasing similarity in these behaviors. In well-preserved areas, we expect the conditions to favor the presence of more specialized species, with more divergent behaviors^31^, such as the species of the genus *Microstigma* Rambur, 1842.

## Material and methods

### Study area

**Figure 1:**
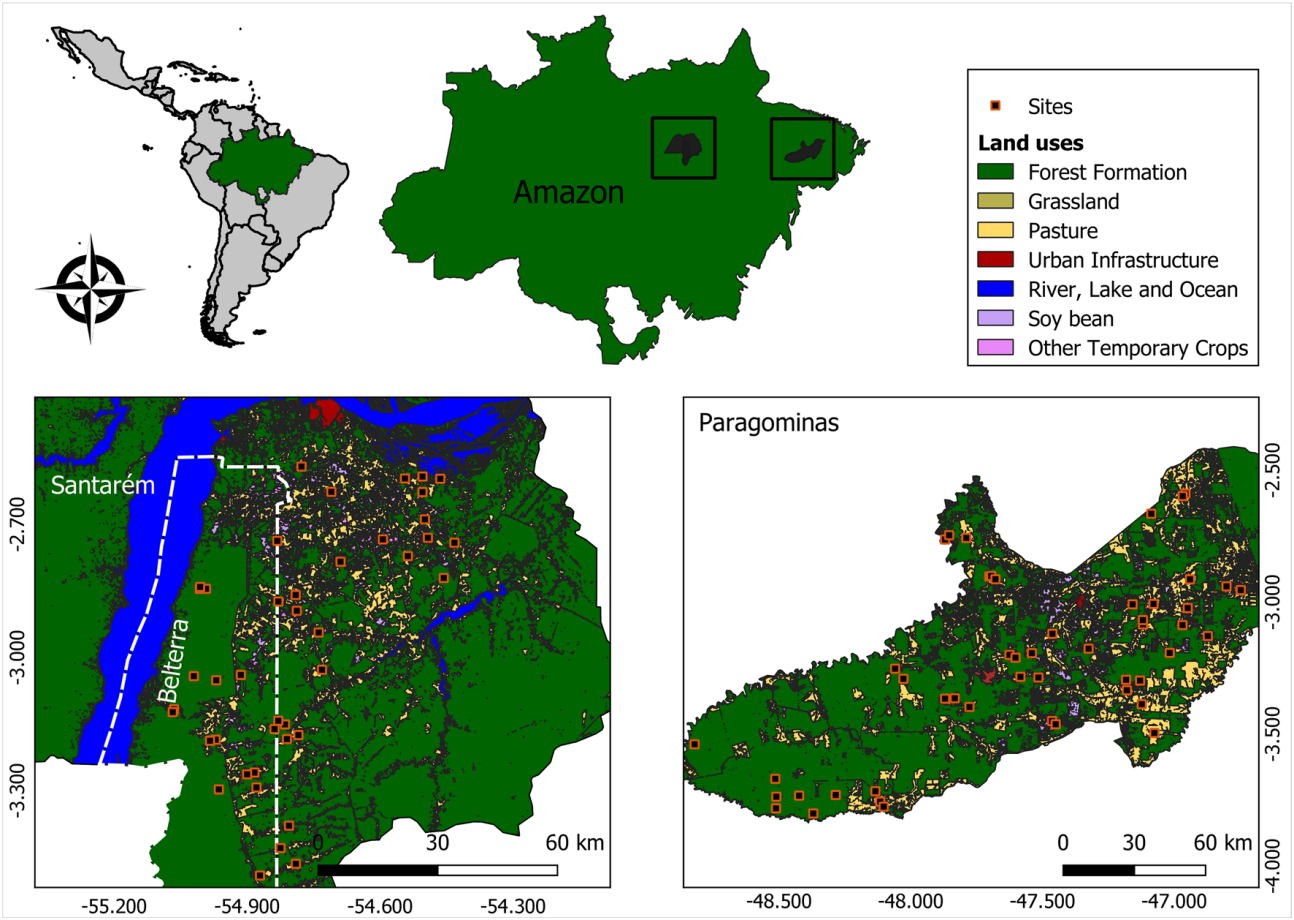
The study areas, located in two regions of the eastern Amazon in the Brazilian state of Pará.

Fieldwork was conducted at 92 streams (first to third order watercourses, in the classification of Strahler^32^), in two different regions of the eastern Amazon (Fig. 1). We collected data at 42 streams in Santarém and Belterra, and 50 streams in Paragominas, all in Pará state (Brazil). Both regions have a humid tropical climate, classified as Af in the Köppen system. The local vegetation is predominantly rainforest, with a few tracts of Amazonian savannah near Santarém. Both regions encompass a gradient of land use, which varies from highly impacted areas—primarily monocultures and pasture—to well-preserved primary forest.

### Biological data

Biological data were collected in both study regions during the dry season only, for four reasons: (1) the ecophysiological requirements of the odonates^6^; (2) to standardize the sampling period and minimize sampling noise in the analyses (see^33^); (3) because a number of previous studies have shown that odonate diversity may be higher during the dry season in the Amazon region, and (4) the reduced depth of the water during this season, which forces the odonates to aggregate at smaller bodies of water, facilitating sampling. During the rainy season, by contrast, conditions typically hamper, or even prohibit altogether the collection of a realistic and representative sample of the odonate communities found on the floodplains of the Amazon^25^.

The species of the suborders Anisoptera and Zygoptera were collected using the “fixed area transect” method or the “Odonate Sampling Protocol” (OSP)^34^. We collected specimens within a total of 20 5-m segments at each stream (100 m of total sampling effort at each site). We captured the specimens using an entomological net while walking along the transect for one hour. Each transect was sampled invariably between 10:00 and 14:00 h, when most of the target species are active, and always on sunny days. We identified all the specimens to the species level, using taxonomic keys and illustrated guides^35–41^, and all the specimens were deposited as vouchers in the collection of the Zoology Museum at the Universidade Federal do Pará (UFPA) in Belém, Brazil.

### Environmental features

We collected environmental data and described the physical habitat, together with the biological data on each stream. We measured a total of 29 environmental variables (see Supplementary Material S1), which have all been used in previous studies and have been shown to be important predictors for the assessment of the effects of different types of land use on odonates^26^. We measured 26 of these variables at each stream following an adapted version of the protocol published by the United States Environmental Protection Agency (US-EPA) and calculated the environmental metrics following^42^. This protocol assesses the characteristics of each stream, providing information on the morphology of the channel, hydraulics, substrates, the availability of shelters for the aquatic biota, the amount and size of woody debris, the cover and structure of the riparian vegetation, and human influences^43^. Two physical and chemical descriptors of the water were also measured, using a multiparametric Horiba device in three equidistant sections of the stream segment (downstream, middle, and upstream). We also calculated the Habitat Integrity Index, HII (see^44^) to provide a score of physical integrity for each study stream. This index is generated using 12 parameters that evaluate different aspects of the morphology of the channel and its surroundings. Values of HII closer to 1 indicate more conserved environments, while those closer to 0 are sites with a high level of degradation^45^. This protocol has been widely used to assess environmental conditions in the Amazon (for more information, see^25,43,46,47^).

### Behavioral traits

We use direct literature classification data and morphological data as proxies to assess behavioral syndromes. This strategy has been widely used with success in studies of functional diversity^48,49^. We categorized the behavioral traits in five classes: (1) territoriality; (2) contest displays; (3) type of oviposition; (4) use of oviposition substrates, and (5) mate-guarding strategies. These categories were defined based on the literature indexed in the Web of Science and Google Scholar databases, using the name of each study species as the keywords (Supplementary Material S2). Given the lack of data for most species, we made every possible effort to complete the categories by consulting specialists on odonate behavior, but even then, some species (in particular, the rarest and most recently-described taxa) lacked some behavioral parameters. In these cases, we obtained information on the behavioral traits of congeneric species, identified the most common behavior in the genus, and extrapolated it to the species lacking data. This strategy has been used successfully to reduce knowledge gaps in a number of previous studies of the odonates^27,29,50^.

We also used five morphological traits as a proxy for dispersal behavior and territoriality: (1) abdomen length; (2) thorax volume; (3) wing stroke; (4) wing load^51^, and (5) the wing–thorax ratio. These variables were obtained by measuring the total length (TL), thorax width (Thw), abdomen length (AL), and the forewing length (FL) and width (FW). The total length (1) was calculated as the distance between the head and the tip of the abdomen, (2) the thorax volume (π radius of the thorax^2^*4/3) provides an index of flight muscle volume, which is a predictor of flight capacity or dispersal distance^52^; (3) the wing stroke (πFL^2^*FW) is proportional to the area of the wing and the amount of air displaced at each stroke of the wings, whose volume is related to πr^2^h (where r represents the length of the wing and h, its width)^53^—this metric also predicts flight performance, and (4) the wing load (thorax volume/wing stroke), for which, we considered the wing stroke to be a proxy of the wing area, based on the formula: thorax volume/wing area, to provide the wing load index. We also calculated (5) the wing–thorax ratio by dividing the squared forewing length by the volume of the thorax, to estimate the allometry of the body. Lower values of this ratio indicate stouter bodies and a capacity for faster flight, whereas higher values indicate slenderer bodies and slower flight^54^. To obtain these measurements, we analyzed specimens deposited in the collection of the UFPA Ecology and Conservation Laboratory in Belém. We selected a total of ten male specimens of each species to obtain the measurements necessary to calculate the parameters described above. To be included in the study, these specimens had to be in good condition, and were selected randomly from the collection, including individuals collected in both degraded and preserved environments. We obtained the morphological measurements only for male individuals, given the reduced abundance of females in the study area, and the lack of taxonomic keys for females. This standard analysis of the male specimens (with all length measures being obtained from the right side of the body) also avoids potential intraspecific differences associated with sexual dimorphism. For species represented by fewer than ten individuals, we measured all the specimens that were in a good condition. All the measurements were obtained in triplicate by three different researchers (to minimize error) using a digital calliper (precision of 0.01 mm), with the mean of these values being considered for analysis.

### Behavioral diversity

We compiled a matrix of ten behavioral traits for each species and converted it into a similarity matrix using the Gower distance^55^ (Supplementary Material S2). We then calculated the FRic, FEVE, and FDiv indices proposed by Villéger^56^. The behavioral richness thus estimates the set of niches occupied by the species that make up a community, while the evenness evaluates the distribution of the insects among the behavioral niches occupied by the different species. Lastly, the behavioral divergence indicates the level of niche differentiation in the community, where the greater the divergence, the more differentiated the community, and thus, the lower the competition for resources. These three indices are important because they quantify relevant aspects of the behavioral diversity of a community in a complementary fashion, with the species distributed in a multidimensional behavioral space^56^.

### Data analysis

We checked for multicollinearity in our environmental data using a variance inflation factor (VIF). This analysis was conducted sequentially, until all the variables presented values of VIF below^57^. Given the differential responses of the two odonate suborders (Anisoptera and Zygoptera) to environmental gradients^25^, we ran the analyses separately for each suborder. We conducted an a priori correspondence analysis (CA) of the abundance matrices, based on the log(x + 1) transformed values, to determine the association of the behavioral traits with environmental features and species. We also ran a weighted mixed multivariate analysis (Hill–Smith analysis), using the morphological and behavioral trait matrices, with the species as the weight and the CA values as the response variable. We then ran a Principal Components Analysis (PCA), which included all the standardized environmental variables and species. Finally, we plotted graphs overlaying the scores of the environmental variables and species with the morphological and behavioral traits.

We ran multiple regressions with forward stepwise model selection to test for the effects of environmental changes on the behavioral diversity of the odonates. For this, the behavioral richness, evenness, and divergence were defined as the response variables. The variables of the physical and limnological structure of the streams were defined as the predictive variables (the metrics we selected for each model are shown in Tables 1 and 2). All the analyses were run in the R environment, using the dbDF, lm, decostand, dudi.coa, dudi.pca, dudi.hillsmith and vif functions of the FD^58^, vegan^59^, Ade4^60^, and faraway^61^ packages.

**Table 1:** Results of the multiple regressions of the relationship between the behavioral diversity metrics (divergence, evenness, and richness) of the Anisoptera and selected environmental variables in two regions of the eastern Amazon, Brazil.

Environmental variable	Beta	Standard error of the beta value	t	p
**Divergence (R**^**2**^** = 0.61)**				
Intercept	0.724	0.014	49.907	< 0.001*
Habitat Integrity Index (V1)	− 0.103	0.021	− 4.992	< 0.001*
Mean small woody debris cover (V5)	0.028	0.021	1.342	0.189
Litter (V6)	0.039	0.025	1.582	0.124
Riparian canopy (V8)	0.112	0.025	4.492	< 0.001*
Mean small tree canopy cover (V9)	0.023	0.022	1.053	0.300
Substrate D50 (V12)	− 0.047	0.017	− 2.744	< 0.001*
Large woody debris in channel (V15)	− 0.089	0.023	− 3.940	< 0.001*
Thalweg depth (V20)	0.030	0.020	1.515	0.140
Substrate of fine sediment (V21)	0.058	0.022	2.614	0.013*
Fine litter (V23)	− 0.024	0.018	− 1.360	0.183
Mean slope of catchment (V26)	0.050	0.018	2.731	0.010*
Secondary forest in riparian network (V28)	− 0.031	0.018	− 1.740	0.091
Intensity of non-forest land use (V29)	− 0.038	0.021	− 1.826	0.077
**Evenness (R**^**2**^** = 0.46)**				
Intercept	0.451	0.022	20.254	< 0.001*
Habitat Integrity Index (V1)	− 0.045	0.026	− 1.702	0.098
Electrical conductivity (V2)	0.098	0.029	3.366	< 0.001*
Dissolved oxygen (V3)	0.035	0.026	1.351	0.185
Litter (V6)	0.126	0.035	3.592	< 0.001*
Mid-stream canopy density (V7)	0.031	0.027	1.145	0.260
Mean slope (%) (V11)	0.056	0.029	1.944	0.054
Pipes, influent and effluent (V13)	− 0.038	0.026	− 1.462	0.153
Large woody debris in channel (V15)	0.031	0.027	1.131	0.266
Volume of wood (V18)	− 0.065	0.034	− 1.915	0.064
Mean catchment slope (V26)	− 0.061	0.028	− 2.180	0.036*
**Richness (R**^**2**^** = 0.56)**				
Intercept	0.032	0.005	6.801	< 0.001*
Habitat Integrity Index (V1)	− 0.031	0.006	− 5.256	< 0.001*
Electrical conductivity (V2)	0.017	0.006	2.787	0.008*
Dissolved oxygen (V3)	0.011	0.005	1.948	0.049*
Mid-stream canopy density (V7)	0.007	0.005	1.398	0.171
Riparian canopy (V8)	0.008	0.007	1.109	0.275
Fast flowing water (V25)	− 0.010	0.006	− 1.722	0.094
Mean catchment slope (V26)	0.008	0.005	1.576	0.124
Secondary forest in riparian network (V28)	− 0.006	0.005	− 1.201	0.238
Intensity of non-forest land use (V29)	− 0.011	0.006	− 1.857	0.072

The codes of the environmental variables are provided in Supplementary Material S2.

*Significant value (p < 0.05).

**Table 2:** Results of the multiple regressions of the relationship between the behavioral diversity metrics (divergence, evenness, and richness) of the Zygoptera and selected environmental variables in two regions of the eastern Amazon, Brazil.

Environmental variable	Beta	Standard error of the beta value	t	p
**Divergence (R**^**2**^** = 0.37)**				
Intercept	0.804	0.010	77.173	< 0.001*
Litter (V6)	− 0.022	0.014	− 1.533	0.130
Mean small tree canopy cover (V9)	0.038	0.015	2.531	0.014*
Mean slope (%) (V11)	0.013	0.012	1.116	0.268
Substrate D50 (V12)	− 0.028	0.012	− 2.456	0.027*
Non-agricultural land use (V14)	0.027	0.012	2.243	0.029*
Volume of wood (V19)	0.019	0.014	1.347	0.183
Substrate of fine sediment (V21)	− 0.014	0.015	− 0.884	0.380
Mean catchment slope (V26)	− 0.021	0.012	− 1.760	0.083
**Evenness (R**^**2**^** = 0.19)**				
Intercept	0.584	0.021	27.920	< 0.001*
Habitat Integrity Index (V1)	0.050	0.023	2.163	0.0340*
Litter (V6)	− 0.041	0.026	− 1.586	0.117
Mid-stream canopy density (V7)	0.041	0.025	1.662	0.101
Large woody debris in channel (V15)	0.059	0.024	2.459	0.016*
Volume of wood (V19)	0.032	0.024	1.336	0.186
**Richness (R**^**2**^** = 0.33)**				
Intercept	0.112	0.007	16.353	< 0.001*
Habitat Integrity Index (V1)	0.017	0.010	1.806	0.075
Electrical conductivity (V2)	0.032	0.009	3.611	< 0.001*
Litter (V6)	− 0.011	0.009	− 1.221	0.226
Mid-stream canopy density (V7)	0.013	0.008	1.561	0.123
Volume of wood (V19)	0.018	0.009	1.990	0.049*
Intensity of non-forest land use (V29)	− 0.016	0.010	− 1.633	0.107

The codes of the environmental variables are provided in Supplementary Material S2.

*Significant value (p < 0.05).

## Results

### Environmental features

The study streams were located along a gradient of land use. Some of these streams were located within highly impacted environments, with up to 96% of the area of the drainage basin under anthropogenic land use, while others were inserted within well-preserved remnants of Amazon forest (Supplementary Material S1). The HII values ranged from 0.08 to 0.99, with a mean of 0.64 and standard deviation (SD) of 0.20. The canopy openness ranged from 0.0 to 1.0 (mean = 0.85 ± SD = 0.24). The variable that varied most was the substrate with a sediment grain size of D50 (mean = 201.32 mm ± SD = 579.23 mm).

### Biological data

We collected 3107 individuals of 101 odonate species, including 49 anisopterans and 52 zygopterans. The most abundant species were *Erythrodiplax basalis* (Kirby, 1897) (N = 294), *Mnesarete aenea* (Selys, 1853) (N = 261), and *Erythrodiplax fusca* (Rambur, 1842) (N = 200), whereas 18 species were represented by only one individual (Supplementary Material S2). *Micrathyria romani* Sjöstedt, 1918, *Macrothemis ludia* Belle, 1987, *Oligoclada walkeri* Geijskes, 1931, *Phyllogomphoides cepheus* Belle, 1980, and *Oligoclada abbreviata* (Rambur, 1842) were all found in streams with a higher level of conservation, whereas *Oligoclada amphinome* Ris, 1919, *Dasythemis esmeralda* Ris, 1910, *Erythrodiplax paraguayensis* (Förster, 1905), and *Progomphus intricatus* Hagen *in* Selys, 1858 were found in streams with greater environmental disturbance (Supplementary Material S3). We found that the zygopteran species *Argia infumata* Selys, 1865, *Heteragrion aurantiacum* Selys, 1862, *Heliocharis amazona* Selys, 1853 and *Epipleoneura capilliformis* (Selys, 1886) were characteristic of better preserved streams, while *Argia reclusa* Selys, 1865, *Acanthagrion kennedii* Williamson, 1916, *Neoneura rubriventris* Selys, 1860, and *Acanthagrion jessei* Leonard, 1977 were observed at disturbed streams (Supplementary Material S4).

### Behavioral features

**Figure 2:**
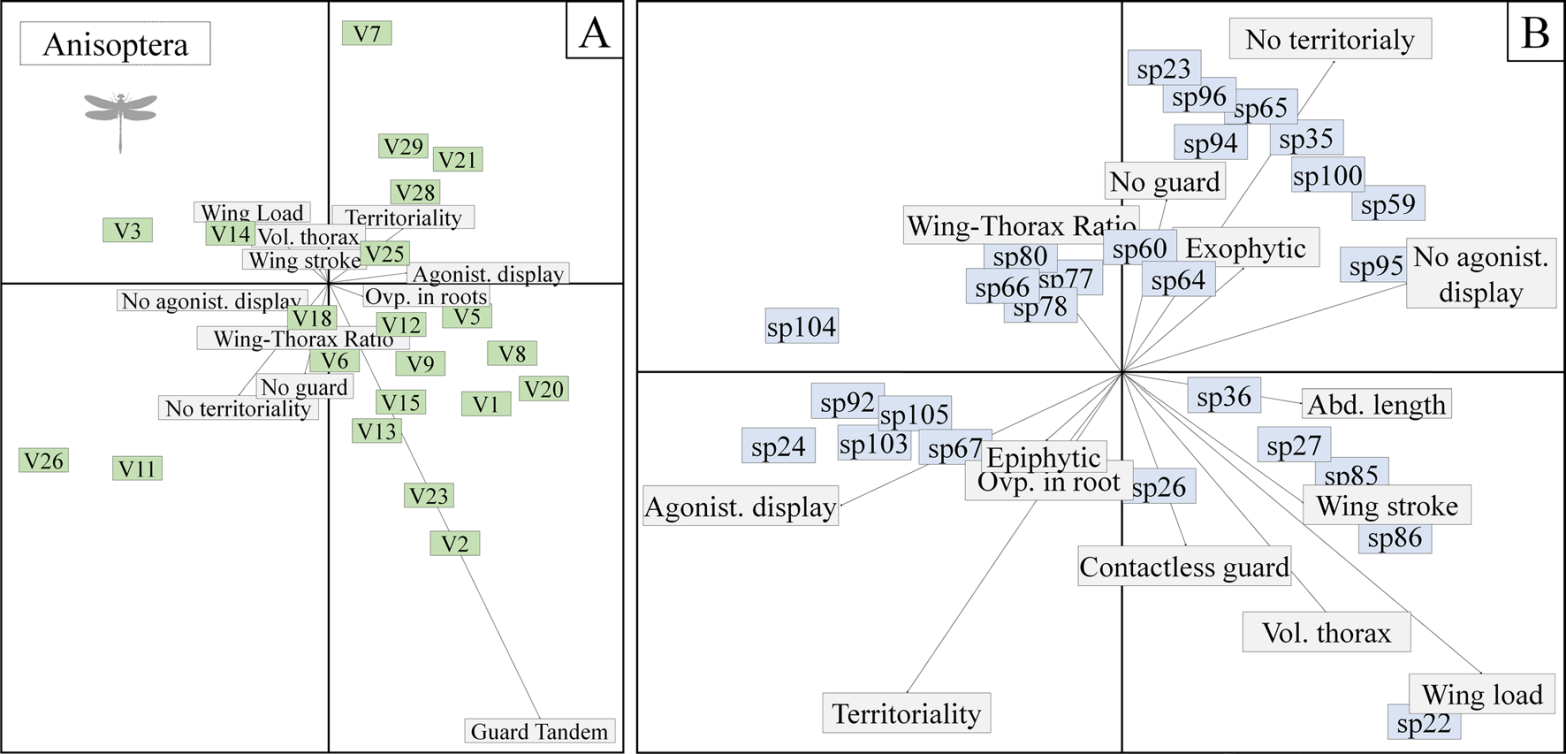
Superimposed ordination of the environmental characteristics and behavioral traits of the species of Anisoptera (**A**) and of the species with these traits (**B**) sampled in two regions of the eastern Amazon. *V1* habitat integrity index, *V2* electrical conductivity, *V3* dissolved oxygen, *V5* small woody debris, *V6* litter, *V11* mean slope, *V12* substrate D50, *V19* volume of wood, *V21* substrate of fine sediment, *V25* fast flowing water, *V26* mean catchment slope, *V28* secondary forest in the Riparian Network, *V29* non-forest land use intensity at the site.

The observed variation in the environment had a range of effects on the behavioral traits of the anisopterans. Species with tandem oviposition (e.g., *Rhodophygia cardinalis* Erichson in Schomburgk, 1848) covaried positively with fine litter substrates (V23) and electrical conductivity (V2). Non-territorial and non mate-guarding species covaried positively with the slope of the hydrographic basin (V11) and the amount of litter (V6). By contrast, territoriality was associated positively with substrates of fine sediments (V21), secondary riparian forest (V28), and the intensity of the non-forest land use at a local scale (V29). Wing load and thorax volume covaried positively with non-agricultural land use (V14) (Fig. 2a). Considering the relationship between the species and their behavioral traits, the behavioural repertoires of *Rhodophygia cardinalis, Dasythemis esmeralda, Progomphus intricatus, Micrathyria pseudeximia* Westfall, 1992*, Erythemis credula* Hagen, 1861*, Phyllocycla bartica* Calvert, 1948, and *Cacoides latro* Erichson, 1848 may have been the most affected, given that the alterations of environmental variables have a greater influence on the behaviors of these species (Fig. 2b).

**Figure 3:**
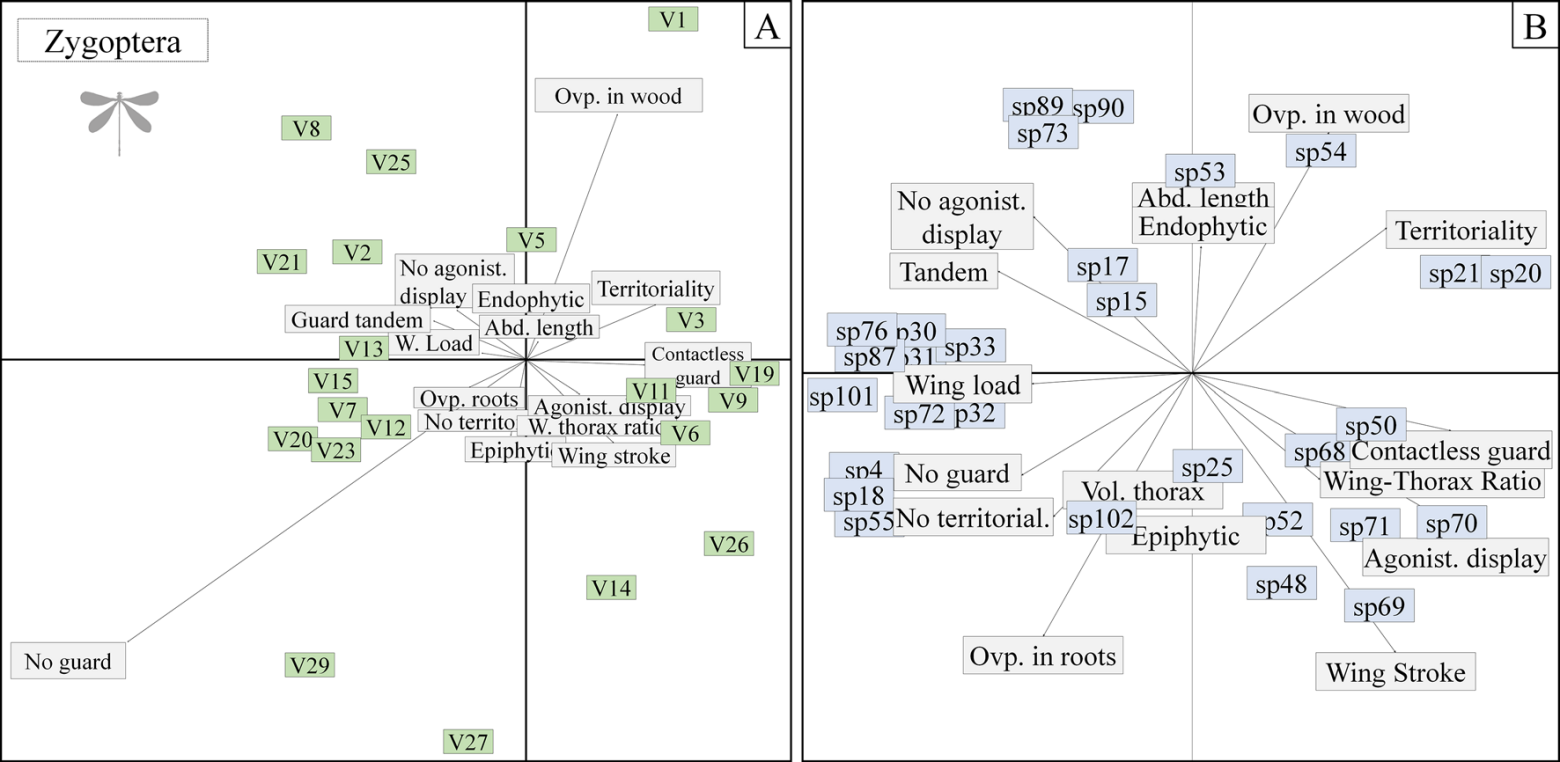
Superimposed ordination of the environmental characteristics and behavioral traits of the species of Zygoptera (**A**) and of the species with these traits (**B**) sampled in two regions of the eastern Amazon. *V1* habitat integrity index, *V2* electrical conductivity, *V3* dissolved oxygen, *V5* small woody debris, *V6* litter, *V11* mean slope, *V12* substrate D50, *V19* volume of wood, *V21* substrate of fine sediment, *V25* fast flowing water, *V26* mean catchment slope, *V28* secondary forest in the Riparian Network, *V29* non-forest land use intensity at the site.

In the zygopterans, we observed that the species which oviposit on decayed wood in the streams (e.g., those of the genus *Chalcopteryx*) covaried positively with the HII (V1). Tandem mate guarding and the absence of agonistic displays covaried positively with conductivity (V2) and substrates with fine sediments (V21). Territoriality was correlated positively with dissolved oxygen (V3), while non-contact mate guarding was correlated positively with dissolved oxygen (V3), the volume of woody debris in the channel (V19), the mean small tree cover (V9), and the mean slope (V11). In turn, species with no mate guarding behavior covaried positively with the intensity of the non-forest land use at a local scale (V29) (Fig. 3a). Considering the relationships between the species and the behavioral traits, the behavioral repertoires of *Acanthagrion adustum* Williamson, 1916*, Chalcopteryx radians* Ris, 1914, *Heteragrion icterops* Selys, 1862*, Acanthagrion ascendens* Calvert, 1909 and *Argia tupi* Calvert, 1909 may have been the most affected, given that the alterations in the environmental variables had a greater influence on their behaviors (Fig. 3b).

### Environmental features and behavioral diversity

**Figure 4:**
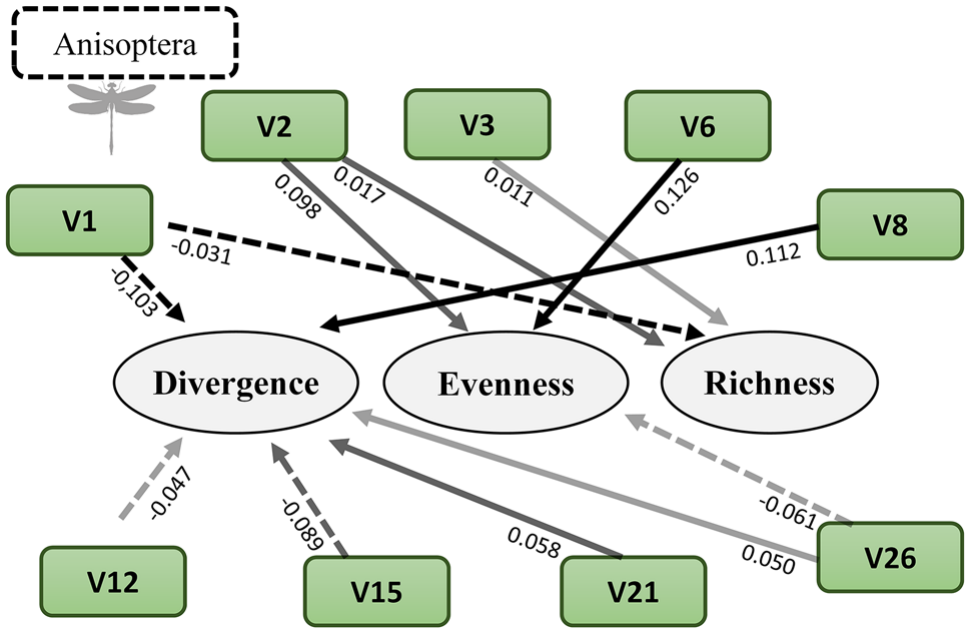
Relationships between the environmental variables and the behavioral diversity metrics (divergence, evenness and richness) in the Anisoptera. The continuous lines represent positive relationships and the dashed lines, negative ones. The darker the arrow, the higher the beta value. The values presented refer to the beta values of multiple regressions. *V1* habitat integrity index, *V2* electrical conductivity, *V3* dissolved oxygen, *V6* litter, *V8* riparian canopy, *V12* substrate D50, *V15* large woody debris in channel, *V21* substrate of fine sediment, *V26* mean catchment slope.

When we analyzed the impact of environmental features on behavioral diversity, we found that the environment explained 61% of the behavioral divergence of the anisopterans. We highlight the positive relationship between behavioral divergence and riparian canopy cover (V8), and the negative relationship with the HII (V1) and large woody debris in the channel (V15). Environmental variables explained 46% of the behavioral evenness, having a positive relationship with conductivity (V2) and litter (V6), and a negative relationship with the slope of the catchment (V26), and 56% of the behavioral richness, having a negative relationship with the HII (V1) (Table 1; Fig. 4).

**Figure 5:**
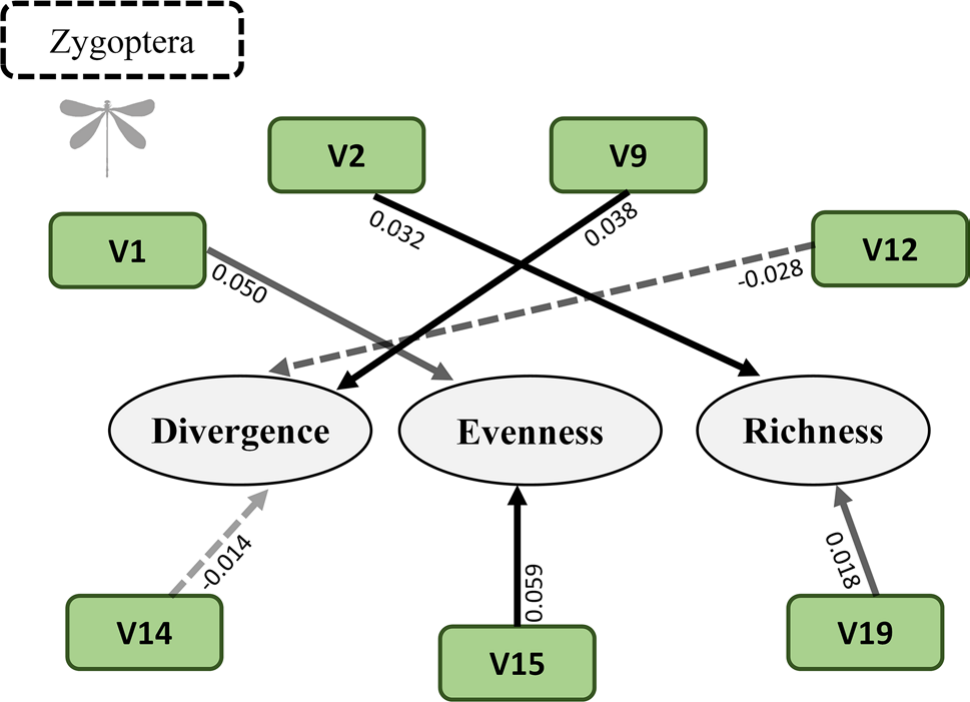
Relationships between environmental variables and the behavioral diversity metrics (divergence, evenness and richness) in the Zygoptera. The continuous lines represent positive relationships and the dashed lines, negative ones. The darker the arrow, the higher the beta values. The values presented refer to the beta values of multiple regressions. *V1* habitat integrity index, *V2* electrical conductivity, *V9* mean small tree canopy cover, *V12* substrate D50, *V14* non-agricultural land use, *V15* large woody debris in channel, *V19* volume of wood.

In the case of the zygopterans, environmental features explained 37% of the behavioral divergence, having a positive relationship with small tree canopy cover (V9), and 19% of the behavioral evenness, with high and positive relationship with the HII (V1) and the large woody debris in the channel (V15), and lastly, 33% of the behavioral richness, also having a positive relationship with conductivity (V2) (Table 2; Fig. 5).

## Discussion

The results of the present study support our hypothesis that the behavioral diversity of the insects of the order Odonata is affected by environmental variables, in particular those related to changes in land use. In the case of the suborder Anisoptera, areas with greater riparian canopy cover, reduced environmental integrity, and the smallest amount of large woody debris in the channel presented a greater divergence of behavioral traits, while areas with higher electrical conductivity and more litter had greater behavioral evenness. Areas of reduced environmental integrity were the richest in behavioral traits. In the Zygoptera, we found that areas with a greater canopy cover of small trees were more behaviorally divergent, i.e., they were occupied by communities composed of species with more varied behavior. Areas with high environmental integrity and larger amounts of large woody debris in the channel had greater behavioral evenness, that is, a greater similarity among individual behavioral strategies, whereas sites with higher electrical conductivity were behaviorally richer.

Recent studies in the Amazon have highlighted the importance of environmental filters for the structuring of odonate communities^62^. Behavioral diversity may provide an indicator that has the potential to contribute to the understanding of the effects of variation in the environment on the species composition of local comunities. Changes in the environment, in particular those provoked by human activities, would thus affect the occurrence of species with given behavioral traits in odonate communities^20,23,29^.

We found that territorial and reproductive behaviors (i.e., type of oviposition and mate-guarding behavior) were closely related to environmental features (physical and chemical variables, related to conservation status of the stream and the presence of vegetation cover). Relationships of this type were expected because oviposition strategies are often linked directly to the amount and quality of the available perches and other resources necessary for oviposition^10,29^. The removal of the riparian vegetation has a marked effect on most zygopteran species, leading to the local exclusion of the species that requires woody substrates within or adjacent to the channel for oviposition^27,29^. This effect is even more noticeable in species with endophytic oviposition, in particular those that oviposit on specific types of aquatic or semi-aquatic plants, such as macrophytes (e.g., *Eleocharis* spp. and *Pontederia parviflora*)^10^. The effect may also be prominent in species with epiphytic oviposition, which relies on a certain degree of heterogeneity in the oviposition substrates within the stream (e.g., rocky surfaces, decaying wood, roots, leaves, and debris)^63^. The absence of any clear relationship in the species with exophytic oviposition was expected, however, because this type of oviposition depends only on the availability of water^6^. The same reasoning can be applied to territoriality, given that territorial males defend perches with certain specific environmental characteristics and the availability of the resources necessary for the females to oviposit, such as the incidence of sunlight, proximity to the stream, and perch density^16^. Given this, environmental degradation and deforestation will likely select against territorial behavior, reducing its frequency or even excluding it altogether from impacted streams^64^.

In the Anisoptera, behavioral divergence was greatest at sites with both greater dense-canopy riparian vegetation cover and lower environmental integrity. However, behavioral richness was higher only at the sites with less intact environments. Evenness was highest at sites with higher electrical conductivity and larger amounts of litter. Anisopterans are larger than zygopterans, in general, and thus have a lower body surface:volume ratio, and require direct sunlight on their bodies to ensure activity (heliotherms)^6^. Most anisopterans have a greater dispersal capacity in comparison with most zygopterans, however, and have a greater dietary amplitude, and more generalist behavior^65^. The larger body size and dispersal capacity of the anisopterans may be reflected in the more intense interspecific competition observed in this suborder, which would mean that, for its species to coexist, they may have to be more divergent, to avoid niche overlap. The greater morphological similarities of the coexisting species may reflect either a lack of niche specialization^66^ or simply the fact that these species are more generalist. Many odonate species (most zygopterans) require habitats with specific characteristics^6,67^, while others (most anisopterans) occur in varying environments and are able to exploit the different aquatic habitats available along the course of a stream^68^. Obviously, however, considerable variation is found within each suborder, or even family or genus, which limits the potential for the reliable extrapolation or generalization of these patterns. In this case, basic studies of the biology of odonate species should be the principal priority at the present time, with more ample analytical approaches, which aim to identify general trends among species, independent of their suborder, in a manner similar to the approach of Bastos et al.^46^.

The greatest behavioral richness was found in environments with reduced habitat integrity, which can be explained by the fact that these environments favor habitat generalist species (e.g., heliophiles) and the local extinction of species specialized for forested environments, species that are more dependent on the adequate conservation of environments^25^. We thus expected the observed increase in the number of behavioral traits recorded in degraded areas—which are usually more open habitats—as a result of the behavioral gap left by the absence of the more susceptible species which inhabited these areas previously. The observed pattern of behavioral uniformity may be explained by the fact that the sites that have larger amounts of litter and higher electrical conductivity may also have fewer other types of substrate for oviposition, which may limit the potential for behavioural variation in these environments.

We found evidence that some zygopteran behaviors are highly dependent on the presence of riparian vegetation^10,29^, and are thus correlated with environmental integrity and heterogeneity^3^. As mentioned above, endophytic oviposition requires adequate substrates, and territorial behavior is highly dependent on specific resources for oviposition, and perch density and quality, as well as being influenced by the local density of both males and females^11^. We would thus expect the greater behavioral divergence observed in areas with greater small tree canopy cover to be related to the reduced availability of resources in these areas, given that environmental shifts can modify habitats or conditions in a way that may exclude species with certain behavioral repertoires and favor other taxa with more specific behaviors. As certain environmental conditions may favor specific behavioral patterns to the detriment of others, any shift in these conditions may increase the behavioral differentiation of the local species. The sites with higher electrical conductivity had greater behavioral richness. In the Amazon, streams with high electrical conductivity tend to have more resources for predatory aquatic larvae by favoring the density of algae and, consequently, that of benthic macroinvertebrates^69^. This increased availability of resources may favor the establishment of more specialized species and result in an increase in the behavioral richness of the local zygopterans.

The higher behavioral evenness found in the more preserved environments, and in particular in those with more large woody debris in the channel, may be accounted for primarily by the diversity of resources. In impacted environments, resources tend to be less stable and distributed more unevenly, which may contribute to reduced behavioral evenness and even a lack of equilibrium in the abundance of individuals with diverse behavioral traits. Impacts provoked by shifts in land use, such as deforestation, may affect the distribution of species and their specific behavioral traits. In fact, the loss of certain types of perches and oviposition resources may have a marked effect on the expression of certain types of behavior^27,29^. Impacts caused by dams, such as changes in water flow patterns, may also limit the species with exophytic oviposition that prefer to oviposit in fast-flowing water^6^.

Our results indicate that behavioral diversity would be a valuable metric for studies of environmental impact. This diversity may provide important insights into the mechanisms that determine the differential effects of environmental impacts on different odonate species. We would recommend that future studies amplify the application of metrics that incorporate behavioral parameters. In the specific case of the odonates, the gomphids and aeshnids are of particular interest, due not only to their crepuscular habits and elusive behavior, which limits data collection^6^, but also because of the general lack of behavioral data and information on the conservation status of most species. A number of studies have now focused on species endemic to the Amazon, and the impacts of deforestation and oil palm plantations on their diversity^70^. However, the general lack of behavioral data on the species from the Amazon and other tropical regions, represents a knowledge gap that must be overcome to ensure more conclusive analyses. One possible approach here would be the systematic interpretation of the pressure of environmental filters on the behavioral and functional diversity of these organisms in forest remnants. Although there is an increasing body of knowledge on the behavioral diversity and conservation of the Odonata^29^, this field of research is still incipient. In the present study, we aimed to provide a novel contribution to the understanding of the behavioral ecology and conservation of one of the biologically most diverse regions of our planet, which is currently under threat from a wide range of anthropic pressures.

## Supplementary Information

Supplementary Information.
