# Supplementary material for: Impact of environmental changes on the behavioral diversity of the Odonata (Insecta) in the Amazon
Source: Sci Rep. 2021 May 7;11:9742. doi: 10.1038/s41598-021-88999-7 (PMC8105400; doi:10.1038/s41598-021-88999-7)
Supplement: Supplementary file 1 — Supplementary Information. [file 41598_2021_88999_MOESM1_ESM.docx]

**Impact of environmental changes** **on the behavioral diversity of the Odonata (Insecta) in the Amazon**

Bethânia O. de Resende^1,2*^, Victor Rennan S. Ferreira^1,2^, Leandro S. Brasil^1^, Lenize B. Calvão^2^, Thiago P. Mendes^1,6^, Fernando G. de Carvalho^1,2^, Cristian C. Mendoza-Penagos^1^; Rafael C. Bastos^1,2^, Joás S. Brito^1,2^, José Max B. Oliveira-Junior ^2,3^, Karina Dias-Silva^2^; Ana Luiza-Andrade^1^; Rhainer Guillermo^4^; Adolfo Cordero-Rivera^5^ & Leandro Juen^1,2^

Supplementary material

**S1.** Mean and standard deviation of the environmental variables of the streams sampled in two regions of the Eastern Amazon, Brazil.

| **Variables** | **Variable name** | **Means** | **Standard deviation** |
| --- | --- | --- | --- |
| V1 | Habitat Integrity Index | 0.64 | 0.20 |
| V2 | Conductivity | 25.04 | 12.45 |
| V3 | Dissolved oxygen | 5.23 | 1.40 |
| V4 | Mean natural cover | 54.81 | 37.77 |
| V5 | Mean small woody debris areal cover | 14.03 | 11.93 |
| V6 | Litter X | 9.89 | 17.36 |
| V7 | Standard deviation of canopy density mid-stream (%) | 12.38 | 10.65 |
| V8 | Riparian canopy presence % | 0.85 | 0.24 |
| V9 | Mean small trees canopy cover - > 5 m high and < 0.3m DBH | 34.07 | 27.09 |
| V10 | Dossel + sub % | 0.88 | 0.19 |
| V11 | Mean slope (%) | 0.44 | 0.46 |
| V12 | Log10 Substrate D50 | 201.32 | 579.24 |
| V13 | Pipes, influent and effluent (proximity-weighted index) | 0.10 | 0.17 |
| V14 | Non-agricultural types | 0.12 | 0.28 |
| V15 | LWD volume in active channel (m3/reach) | 1.13 | 1.80 |
| V16 | Number of wood + sup | 1.20 | 1.79 |
| V17 | Number of wood/100 + sup | 7.97 | 11.90 |
| V18 | Volume of wood | 0.15 | 0.24 |
| V19 | Volume of wood/100 | 0.97 | 1.63 |
| V20 | Standard deviation of thalweg depth (cm) | 14.62 | 8.82 |
| V21 | Standard deviation of substrate embedded in fine sediments - channel + margin (%) | 31.68 | 9.64 |
| V22 | Coarse gravel and larger 16mm diametric (%) | 55.36 | 51.10 |
| V23 | % fine litter (NEW) | 3.42 | 6.65 |
| V24 | Slow flow - glides + all pool types (%) | 46.07 | 44.11 |
| V25 | Fast flow - falls + cascades + rapids + riffles (%) | 8.38 | 16.43 |
| V26 | Catchment mean slope (%) | 5.87 | 2.72 |
| V27 | % Secondary Forest at Catchment buffer scale | 16.04 | 15.43 |
| V28 | % Secondary Forest at Riparian Network 100m buffer scale | 19.27 | 18.67 |
| V29 | Land use intensity (Non forest) at Local 100m buffer scale | 0.35 | 0.33 |

**S2.** Behavioral and morphological traits and general abundance of Odonata species, sampled in two regions of the Eastern Amazon, Brazil. Ab= abundance. Y= Yes; N= No; Epi= Epiphytic; Endo= Endophytic; Exo= Exophytic.

| **Suborders/Species** | **Territoriality** | **Fight**  **display** | **Oviposition types** | **Oviposition substrate** | **Oviposition guard** | **Abdomen**  **length** | **Volume**  **thorax** | **Wing**  **stroke** | **Wing**  **load** | **Wing thorax**  **ratio** | **Ab** |
| --- | --- | --- | --- | --- | --- | --- | --- | --- | --- | --- | --- |
| **Anisoptera** |  |  |  |  |  |  |  |  |  |  |  |
| *Argyrothemis argentea* | Y | N | Exo | Surface | contact | 22.32 | 8.811 | 9,748 | 0.001 | 68.643 | 25 |
| *Cacoides latro* | Y | N | Epi | Roots | contact | 54.54 | 240.785 | 48,091 | 0.005 | 7.474 | 2 |
| *Dasythemis esmeralda* | N | N | Exo | Surface | contact | 17.12 | 3.181 | 11,523 | 0.000 | 206.183 | 9 |
| *Diastatops obscura* | Y | Y | Epi | Roots | contact | 16.34 | 6.293 | 10,256 | 0.001 | 78.846 | 114 |
| *Elasmothemis cannacrioides* | Y | N | Epi | Roots | contact | 26.12 | 35.179 | 21,380 | 0.002 | 28.518 | 10 |
| *Elasmothemis williamsoni* | Y | N | Exo | Surface | contact | 31.06 | 64.246 | 37,761 | 0.002 | 23.906 | 1 |
| *Erythemis credula* | N | N | Exo | Surface | without_guarding | 24.61 | 25.136 | 12,793 | 0.002 | 27.970 | 2 |
| *Erythemis haematogastra* | Y | N | Exo | Surface | without_guarding | 32.92 | 56.655 | 27,452 | 0.002 | 21.285 | 7 |
| *Erythemis vesiculosa* | Y | N | Epi | Roots | without_guarding | 37.00 | 55.663 | 40,201 | 0.001 | 27.612 | 6 |
| *Erythrodiplax amazonica* | Y | N | Exo | Surface | contact | 18.33 | 16.264 | 10,308 | 0.002 | 37.990 | 24 |
| *Erythrodiplax avittata* | Y | N | Exo | Surface | contact | 11.68 | 6.041 | 5,900 | 0.001 | 69.431 | 2 |
| *Erythrodiplax basalis* | Y | N | Exo | Surface | contact | 17.49 | 8.305 | 6,639 | 0.001 | 54.462 | 294 |
| *Erythrodiplax castanea* | Y | N | Exo | Surface | contact | 18.10 | 11.015 | 10,995 | 0.001 | 57.393 | 2 |
| *Erythrodiplax fusca* | Y | N | Exo | Surface | contact | 17.15 | 10.566 | 8,584 | 0.001 | 52.039 | 200 |
| *Erythrodiplax juliana* | Y | N | Exo | Surface | contact | 18.96 | 13.919 | 10,435 | 0.001 | 44.009 | 12 |
| *Erytthrodiplax ochracea* | Y | N | Exo | Surface | contact | 17.08 | 13.572 | 11,140 | 0.001 | 44.154 | 1 |
| *Erythrodiplax paraguayensis* | Y | N | Exo | Surface | contact | 14.17 | 12.632 | 4,121 | 0.003 | 23.940 | 3 |
| *Fylgia amazonica* | Y | N | Exo | Surface | contact | 13.16 | 3.218 | 3,041 | 0.001 | 93.464 | 6 |
| *Macrothemis ludia* | Y | N | Exo | Surface | contact | 27.09 | 6.705 | 9,460 | 0.001 | 78.960 | 1 |
| *Miathyria marcella* | N | N | Exo | Surface | without_guarding | 25.94 | 52.618 | 21,452 | 0.002 | 18.417 | 1 |
| *Miathyria simplex* | Y | N | Exo | Surface | without_guarding | 19.83 | 9.850 | 12,762 | 0.001 | 64.678 | 3 |
| *Micrathyria aequalis* | Y | N | Exo | Surface | contact | 17.07 | 6.620 | 6,140 | 0.001 | 61.030 | 2 |
| *Micrathyria artemis* | Y | N | Exo | Surface | contact | 23.09 | 22.883 | 15,617 | 0.001 | 36.838 | 12 |
| *Micrathyria eximia* | Y | N | Exo | Surface | contact | 16.60 | 6.535 | 5,400 | 0.001 | 59.266 | 1 |
| *Micrathyria hesperis* | Y | N | Exo | Surface | without_guarding | 16.32 | 10.074 | 6,510 | 0.002 | 43.238 | 4 |
| *Micrathyria pseudeximia* | N | N | Exo | Surface | contact | 17.33 | 3.647 | 7,680 | 0.000 | 134.424 | 1 |
| *Micrathyria romani* | Y | N | Exo | Surface | contact | 14.91 | 2.617 | 4,501 | 0.001 | 142.642 | 9 |
| *Micrathyria ungulata* | Y | N | Epi | Roots | contact | 17.98 | 6.879 | 7,304 | 0.001 | 68.455 | 2 |
| *Nephepeltia berlai* | Y | N | Exo | Surface | contact | 16.31 | 1.697 | 3,752 | 0.000 | 175.191 | 1 |
| *Nephepeltia flavifrons* | Y | N | Exo | Surface | contact | 16.71 | 1.874 | 3,827 | 0.000 | 163.389 | 1 |
| *Oligoclada abbreviata* | Y | N | Exo | Surface | contact | 16.39 | 6.931 | 7,405 | 0.001 | 66.441 | 58 |
| *Oligoclada amphinome* | Y | N | Exo | Surface | contact | 13.74 | 2.499 | 5,473 | 0.000 | 150.312 | 21 |
| *Oligoclada crocogaster* | Y | N | Exo | Surface | contact | 14.08 | 4.124 | 5,480 | 0.001 | 93.557 | 4 |
| *Oligoclada stenoptera* | Y | N | Exo | Surface | contact | 14.94 | 5.459 | 5,530 | 0.001 | 69.153 | 3 |
| *Oligoclada walkeri* | Y | N | Exo | Surface | contact | 17.17 | 6.384 | 8,171 | 0.001 | 77.554 | 77 |
| *Oligoclada xanthopleura* | Y | N | Exo | Surface | contact | 14.45 | 12.243 | 5,225 | 0.002 | 28.198 | 2 |
| *Orthemis biolleyi* | Y | N | Exo | Surface | contact | 30.37 | 105.580 | 33,081 | 0.003 | 13.090 | 3 |
| *Orthemis discolor* | Y | N | Exo | Surface | contact | 31.26 | 132.171 | 40,359 | 0.003 | 12.290 | 60 |
| *Perithemis cornelia* | Y | N | Exo | Surface | contact | 11.75 | 4.923 | 4,059 | 0.001 | 53.835 | 3 |
| *Perithemis lais* | Y | Y | Exo | Surface | contact | 11.06 | 3.483 | 2,961 | 0.001 | 60.925 | 61 |
| *Phyllocycla bartica* | N | N | Epi | Roots | without_guarding | 31.21 | 13.669 | 10,015 | 0.001 | 43.638 | 3 |
| *Phyllogomphoides cepheus* | N | N | Epi | Roots | without_guarding | 43.23 | 69.421 | 29,658 | 0.002 | 18.690 | 1 |
| *Progomphus intricatus* | N | N | Epi | Roots | without_guarding | 28.59 | 13.299 | 9,329 | 0.001 | 40.839 | 1 |
| *Progomphus maculatus* | N | N | Epi | Roots | without_guarding | 17.33 | 7.886 | 3,556 | 0.002 | 35.195 | 1 |
| *Rhodopygia cardinalis* | N | N | Exo | Surface | tandem | 29.69 | 40.316 | 34,368 | 0.001 | 34.060 | 9 |
| *Zenithoptera fasciata* | Y | Y | Exo | Surface | contact | 14.28 | 8.365 | 6,943 | 0.001 | 46.881 | 2 |
| *Zenithoptera lanei* | Y | Y | Exo | Surface | without_guarding | 13.54 | 5.680 | 6,230 | 0.001 | 63.341 | 42 |
| *Zenithoptera viola* | Y | Y | Exo | Surface | contact | 15.20 | 7.791 | 7,151 | 0.001 | 49.460 | 1 |
| *Zonophora calippus* | N | N | Epi | Roots | contact | 38.81 | 86.123 | 28,924 | 0.003 | 14.732 | 1 |
| **Zygoptera** |  |  |  |  |  |  |  |  |  |  |  |
| *Acanthagrion adustum* | N | N | Endo | Roots | tandem | 19.16 | 0.147 | 941 | 0.000 | 1,236.516 | 26 |
| *Acanthagrion aepiolum* | N | N | Epi | Roots | tandem | 23.33 | 0.400 | 1,631 | 0.000 | 656.445 | 6 |
| *Acanthagrion apicale* | N | Y | Endo | Roots | tandem | 28.06 | 1.521 | 2,590 | 0.001 | 252.823 | 11 |
| *Acanthagrion ascendens* | N | N | Endo | Roots | tandem | 23.70 | 18.433 | 6,080 | 0.003 | 25.541 | 7 |
| *Acanthagrion jessei* | N | N | Endo | Roots | tandem | 20.79 | 0.310 | 1,143 | 0.000 | 655.576 | 1 |
| *Acanthagrion kennedii* | N | N | Endo | Roots | tandem | 23.23 | 0.585 | 2,652 | 0.000 | 458.861 | 21 |
| *Acanthallagma luteum* | N | N | Endo | Roots | tandem | 20.03 | 0.472 | 1,201 | 0.000 | 334.456 | 11 |
| *Argia chapadae* | Y | N | Epi | Roots | tandem | 25.00 | 1.595 | 2,326 | 0.001 | 211.052 | 3 |
| *Argia euphorbia* | Y | N | Endo | Roots | tandem | 24.00 | 0.948 | 2,557 | 0.000 | 344.565 | 8 |
| *Argia insipida* | Y | N | Endo | Roots | tandem | 28.50 | 15.591 | 6,532 | 0.002 | 29.649 | 2 |
| *Argia fumigata* | Y | N | Endo | Roots | tandem | 25.96 | 1.451 | 3,204 | 0.000 | 268.138 | 12 |
| *Argia infumata* | Y | N | Endo | Roots | tandem | 24.35 | 0.634 | 2,358 | 0.000 | 516.154 | 100 |
| *Argia mollis* | N | N | Epi | Roots | tandem | 24.81 | 0.716 | 2,338 | 0.000 | 462.294 | 4 |
| *Argia oculata* | Y | N | Endo | Roots | tandem | 24.91 | 1.054 | 2,649 | 0.000 | 335.503 | 44 |
| *Argia reclusa* | Y | N | Endo | Roots | tandem | 26.72 | 1.802 | 5,033 | 0.000 | 221.688 | 1 |
| *Argia smithiana* | Y | N | Epi | Roots | tandem | 24.82 | 1.534 | 2,452 | 0.001 | 239.483 | 11 |
| *Argia tinctipennis* | Y | N | Endo | Roots | tandem | 23.14 | 0.729 | 2,226 | 0.000 | 407.981 | 105 |
| *Argia tupi* | N | N | Endo | Roots | tandem | 28.00 | 18.807 | 7,973 | 0.002 | 28.128 | 2 |
| *Chalcopteryx radians* | Y | Y | Endo | Wood | contact | 17.37 | 0.383 | 2,295 | 0.000 | 560.001 | 74 |
| *Chalcopteryx rutilans* | Y | Y | Endo | Wood | contact | 21.40 | 0.929 | 4,020 | 0.000 | 343.050 | 143 |
| *Dicterias atrosanguinea* | Y | Y | Epi | Roots | tandem | 24.21 | 2.526 | 4,869 | 0.001 | 206.697 | 33 |
| *Epipleoneura capilliformis* | N | N | Endo | Roots | tandem | 28.28 | 0.300 | 3,574 | 0.000 | 1,169.136 | 123 |
| *Epipleoneura fuscaenea* | N | N | Endo | Roots | tandem | 28.64 | 0.412 | 2,699 | 0.000 | 780.617 | 5 |
| *Epipleoneura haroldoi* | N | N | Endo | Roots | tandem | 26.00 | 1.766 | 2,431 | 0.001 | 148.586 | 29 |
| *Epipleoneura metallica* | N | N | Epi | Roots | tandem | 27.44 | 0.424 | 1,282 | 0.000 | 601.605 | 158 |
| *Epipleoneura pereirai* | N | N | Endo | Roots | tandem | 26.59 | 6.636 | 7,305 | 0.001 | 81.122 | 2 |
| *Epipleoneura spatulata* | N | N | Endo | Roots | tandem | 25.75 | 1.949 | 2,823 | 0.001 | 150.044 | 17 |
| *Epipleoneura westfalli* | N | N | Endo | Roots | tandem | 25.94 | 0.503 | 1,898 | 0.000 | 562.575 | 8 |
| *Heliocharis amazona* | Y | Y | Epi | Roots | tandem | 34.95 | 6.853 | 13,309 | 0.001 | 128.281 | 19 |
| *Hetaerina auripennis* | Y | Y | Endo | Roots | tandem | 31.71 | 2.868 | 9,220 | 0.000 | 212.865 | 38 |
| *Hetaerina indeprensa* | Y | Y | Endo | Roots | contact | 30.13 | 3.528 | 7,806 | 0.000 | 156.087 | 125 |
| *Hetaerina sanguinea* | Y | Y | Endo | Roots | tandem | 39.24 | 9.305 | 15,025 | 0.001 | 89.440 | 10 |
| *Heteragrion aurantiacum* | N | Y | Endo | Wood | tandem | 29.99 | 0.761 | 2,008 | 0.000 | 490.395 | 52 |
| *Heteragrion icterops* | Y | Y | Endo | Wood | tandem | 29.32 | 0.712 | 2,441 | 0.000 | 536.079 | 18 |
| *Ischnura capreolus* | N | N | Epi | Roots | without_guarding | 16.86 | 0.300 | 404 | 0.001 | 353.327 | 1 |
| *Mnesarete aenea* | Y | Y | Epi | Roots | contact | 28.68 | 1.273 | 6,972 | 0.000 | 399.841 | 261 |
| *Mnesarete cupraea* | Y | Y | Epi | Roots | contact | 34.92 | 7.696 | 10,710 | 0.001 | 90.079 | 1 |
| *Mnesarete smaragdina* | Y | Y | Epi | Roots | contact | 30.10 | 1.075 | 8,762 | 0.000 | 531.195 | 114 |
| *Mnesarete williamsoni* | Y | Y | Epi | Roots | contact | 29.33 | 2.132 | 7,521 | 0.000 | 248.869 | 88 |
| *Neoneura denticulata* | N | N | Endo | Roots | tandem | 24.16 | 1.415 | 1,623 | 0.001 | 197.766 | 9 |
| *Neoneura bilinearis* | N | N | Endo | Wood | tandem | 23.61 | 1.047 | 1,664 | 0.001 | 249.166 | 1 |
| *Neoneura joana* | N | N | Endo | Roots | tandem | 23.61 | 0.956 | 1,639 | 0.001 | 273.951 | 2 |
| *Neoneura luzmarina* | N | N | Endo | Roots | tandem | 22.17 | 0.858 | 1,663 | 0.001 | 293.579 | 57 |
| *Neoneura rubriventris* | N | N | Endo | Roots | tandem | 22.70 | 1.017 | 1,715 | 0.001 | 246.156 | 23 |
| *Oxystigma petiolatum* | N | N | Endo | Roots | tandem | 26.01 | 3.880 | 2,904 | 0.001 | 116.259 | 25 |
| *Perilestes kahli* | N | N | Endo | Wood | tandem | 39.56 | 1.923 | 4,486 | 0.000 | 220.621 | 12 |
| *Perilestes solutus* | N | N | Endo | Wood | tandem | 39.94 | 2.045 | 4,827 | 0.000 | 195.730 | 2 |
| *Phasmoneura exigua* | N | N | Endo | Roots | tandem | 27.68 | 0.269 | 1,795 | 0.000 | 1,303.949 | 32 |
| *Psaironeura tenuissima* | N | N | Endo | Roots | tandem | 24.59 | 0.155 | 1,405 | 0.000 | 1,882.154 | 47 |
| *Protoneura tenuis* | Y | N | Endo | Roots | contact | 32.13 | 0.345 | 1,486 | 0.000 | 967.002 | 63 |
| *Telebasis sanguinalis* | N | N | Endo | Roots | tandem | 17.10 | 6.620 | 1,874 | 0.004 | 30.460 | 3 |
| *Tigriagrion aurantinigrum* | Y | Y | Endo | Roots | without_guarding | 16.80 | 0.223 | 653 | 0.000 | 626.287 | 26 |


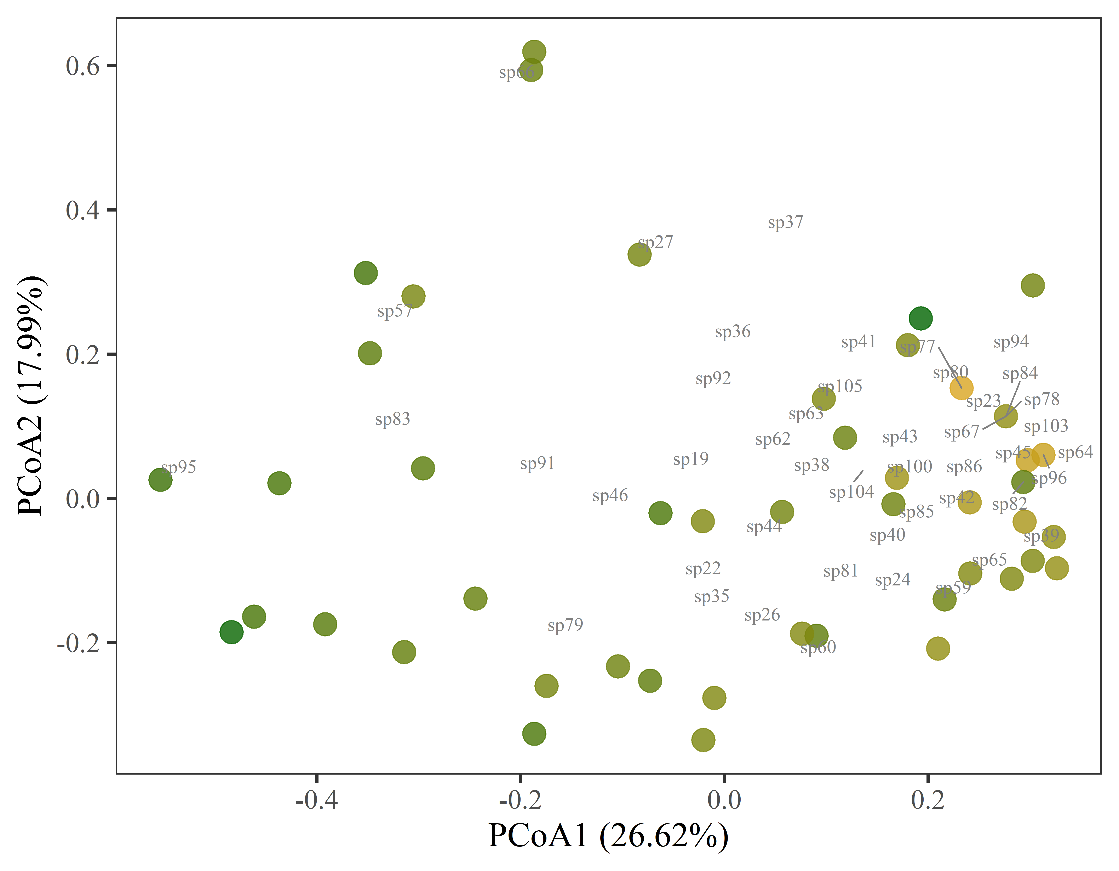


**S3.** Ordination of Anisoptera species through sampling sites. The different shades of color represent the level of conservation of the streams: darker shades indicate more conserved environments, while lighter shades represent points with a higher level of environmental change.

**
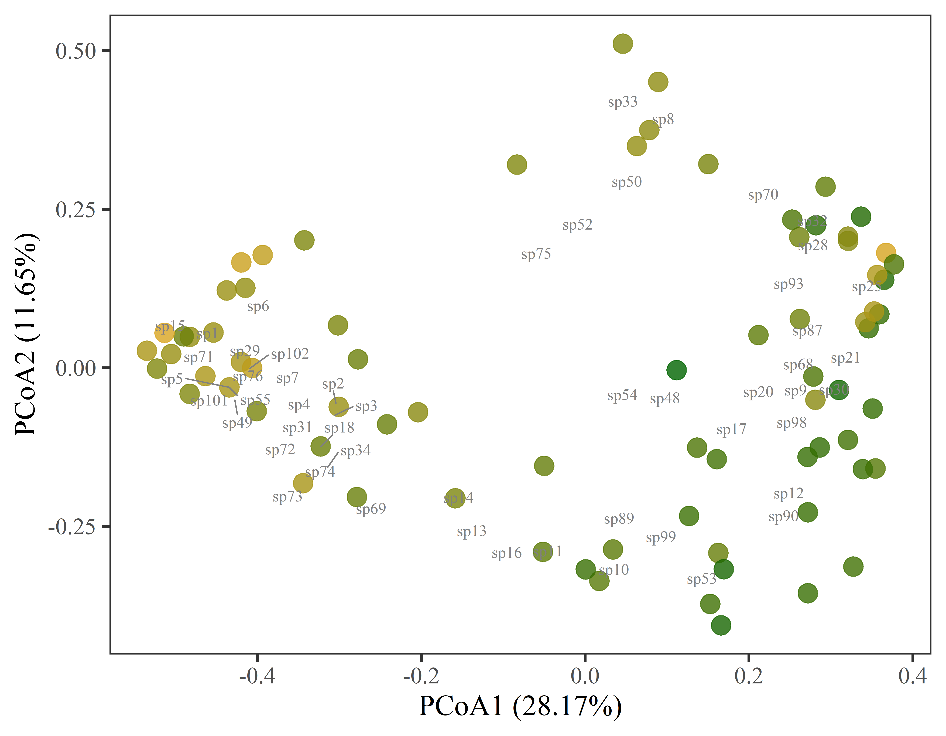
**

**S4.** Management of Zygoptera species through sampling sites. The different shades of color represent the level of conservation of the streams: darker shades indicate more conserved environments, while lighter shades represent points with a higher level of environmental change.
